# Transcriptomics profiling study of breast cancer from Kingdom of Saudi Arabia revealed altered expression of *Adiponectin* and *Fatty Acid Binding Protein4*: Is lipid metabolism associated with breast cancer?

**DOI:** 10.1186/1471-2164-16-S1-S11

**Published:** 2015-01-15

**Authors:** Adnan Merdad, Sajjad Karim, Hans-Juergen Schulten, Manikandan Jayapal, Ashraf Dallol, Abdelbaset Buhmeida, FATIMA AL-THUBAITY, Mamdooh A GariI, Adeel GA Chaudhary, Adel M Abuzenadah, Mohammed H Al-Qahtani

**Affiliations:** 1 Department of Surgery, Faculty of Medicine, King Abdulaziz University, Jeddah, Saudi Arabia; 2 Center of Excellence in Genomic Medicine Research, King Fahad Medical Research Center, King Abdulaziz University, Jeddah, Saudi Arabia; 3 Faculty of Applied Medical Sciences, King Abdulaziz University, Jeddah, Saudi Arabia; 4 KACST Technology Innovation Center for Personalized Medicine at King Abdulaziz University, Jeddah, Saudi Arabia

**Keywords:** Breast cancer, gene expression, microarray, lipid metabolism, Saudi Arabia

## Abstract

**Background:**

Breast cancer incidence rates are increasing at an alarming rate among Saudi Arabian females. Most molecular genetic discoveries on breast cancer and other cancers have arisen from studies examining European and American patients. However, possibility of specific changes in molecular signature among cancer patients of diverse ethnic groups remains largely unexplored. We performed transcriptomic profiling of surgically-resected breast tumors from 45 patients based in the Western region of Saudi Arabia using Affymetrix Gene 1.0 ST chip. Pathway and biological function-based clustering was apparent across the tissue samples.

**Results:**

Pathway analysis revealed canonical pathways that had not been previously implicated in breast cancer. Biological network analysis of differentially regulated genes revealed that Fatty acid binding protein 4, adipocyte (*FABP4*), adiponectin (*ADIPOQ*), and retinol binding protein 4 (*RBP4*) were most down regulated genes, sharing strong connection with the other molecules of lipid metabolism pathway. The marked biological difference in the signatures uncovered between the USA and Saudi samples underpins the importance of this study. Connectivity Map identified compounds that could reverse an observed gene expression signature

**Conclusions:**

This study describes, to our knowledge, the first genome-wide profiling of breast cancer from Saudi ethnic females. We demonstrate the involvement of the lipid metabolism pathway in the pathogenesis of breast cancer from this region. This finding also highlights the need for strategies to curb the increasing rates of incidence of this disease by educating the public about life-style risk factors such as unhealthy diet and obesity.

## BACKGROUND

Breast cancer (BC) is the most common cancer that affects females worldwide and is the second most frequent cause of cancer-related deaths among women in the United States [1]. According to the National Cancer Registry in the Kingdom of Saudi Arabia (KSA), BC was ranked as the most prevalent form of cancer among females, accounting for 25.1% of all newly diagnosed female cancers (5,205) in the year 2009 [2]. The ASR was 22.7/100,000 for the female population. While the median age at presentation is around 63 years in the United States and Western Europe, the median age at presentation in the KSA is 48 years [2]. The genetic variability between patients and tumors drive this clinically heterogeneous disease [3]. Much of the knowledge on the molecular genetics on BC and other cancers has been resulting from examining European and US patients. However, growing scientific knowledge suggests the likelihood of variability in molecular signature between cancers from patients of different ethnic groups. Clear differences in the patterns of p53 mutations in BC were found between Midwest US Caucasian, African- American, Austrian, and Japanese women [4,5]. Besides, varying patterns and the occurrence of germline mutations in BRCA1 and BRCA2 between ethnic groups were also reported [6]. Recently, National Cancer Institute’s Surveillance, Epidemiology, and End Results (SEER) program show that the age-adjusted BC frequency among minority groups are significantly lower than those among white women [7] Increase in BC mortality was also found in African American women when compared to white women [8]. All these differences continue to be mostly unexplained [9]. Moreover, changes in occurrence and clinical characteristics linked with ethnicity or race has got only limited consideration [10]. The current lack of measures to aid in stratifying BC treatment indicates the necessity for a new methodology to formulate better prognostication and therapy prediction. Although the parameters such as histological grade, stage, and tumor size are accepted as prognostic markers for BC [11], about 50% of the patients with ER-positive cancer fail on tamoxifen treatment [12,13].

Transcriptomics approaches that allow screening of thousands of genes in an experiment, has had a major impact on BC research over the past 10 years [14] Several groups have carried out gene expression profiling of BC and classified clinically distinct subclasses of tumors [14], hereditary BC [15], and treatment prediction [16]. Many microarray studies so far reported have led to the discovery of several genes associated with BC. However, most of the gene expression profiling studies has been carried out mainly in the Caucasian population, and inclusion of the non-Caucasian population has been minimal [17,18]. Significant risk factors of BC in the western countries such as nulliparity, low parity, first time pregnancy at late age, having no history of breast feeding, etc., are usually not practiced in the Saudi society, yet BC incidence is still high among women from the KSA [19]. In addition, a study of Taiwanese population reported that women who had more than three full-term pregnancies; first full-term pregnancy at the age of below 30; and three or more years of breast feeding showed significantly reduced risk of BC [20].

These studies suggest that there is an important need to better understand the molecular mechanisms underlying BC from different populations. In view of the existence of genetic differences across regional or geographical locations, more data specific to indigenous populations are needed for comparative analysis of molecular changes in breast tumors among Saudi population. More comprehensive molecular and genomic analyses of breast cancers are important to understand the complexity and severity of the disease and to find ‘druggable’ molecular targets for the development effective treatments. In order to obtain relevant target genes associated with BC patients in the KSA, we aim to start comprehensive microarray-based gene expression profiling study of BC in the Kingdom. Transcriptomic analysis coupled with functional and pathway analysis could lead to new insight into biomarkers and signatures associated with the disease. Deregulated signaling pathways are thought to drive functional processes such as cell growth, cellular proliferation, and invasion of cancer cells. Thus, identifying such underlying driving changes will be vital for studies of tumor progression, for the identification of novel therapeutic targets.

To this end, this study aimed to survey the genes that are differentially regulated in forty five freshly frozen BC tissue samples compared to eight normal controls. The ethnic group of the cohort in this study provides unique dimension to BC research. We examined genome-wide gene expression in order to elucidate the molecular mechanisms underlying malignancy in breast tissues. Based on the differential expression signature, we found biological functions and pathways that are significantly altered in BC. Pathway-based clustering was apparent across the tissue samples. Pathway analysis revealed canonical pathways that have not been implicated in BC, previously. We also demonstrated additional utility of our BC signature by comparative analysis between the USA and the KSA samples reveals marked biological differences in signature. Furthermore, Connectivity Map (cmap) analysis offered hypotheses regarding potential treatments for differential regulation observed in the BC [21]. Thus, our finding might open up new avenues of BC research and assist discover possible new targets for BC treatment where ethnicity plays major role.

## RESULTS

**Figure 1:**
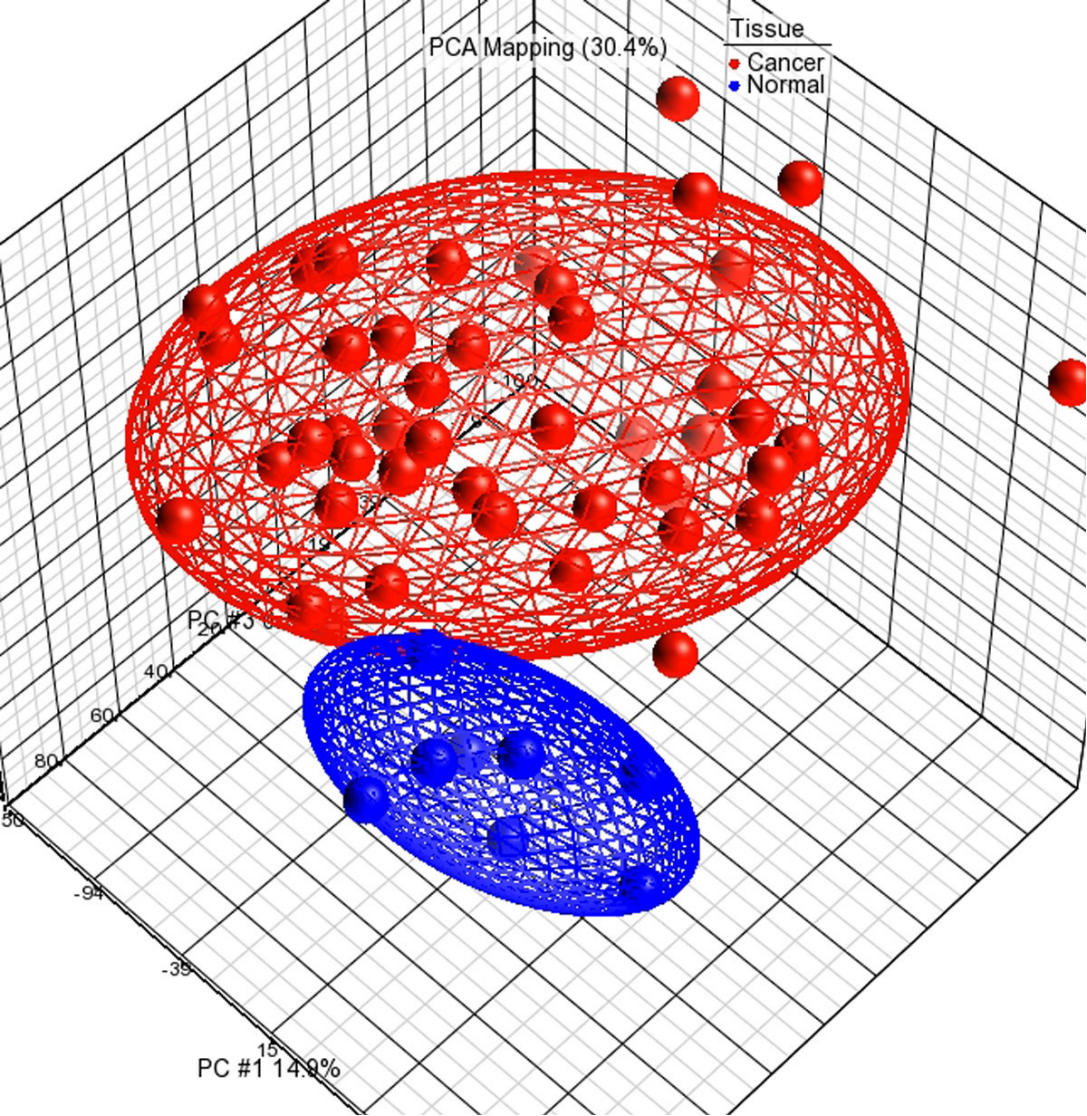
**Principal component analysis of transcriptomic data set.** Description: The top three principal components are plotted on the *X*-, *Y*-, and *Z*-axes, respectively. Overall variation between cancer and normal, where each spot represents an individual array, can be seen by the clustering within each tissue type and the separation between the different tissue types.

The main focus of this study was to determine the transcriptomic profiles of BC representing the Saudi society. To identify genes that are involved in BC development, we analyzed the transcriptomic profiles of nearly 28,000 annotated genes. We profiled forty five fresh breast tissue specimens and compared them with eight normal control samples. We found 48 years as average age of incidence for BC in Saudi Arabia. We also found that 73.3% of BC patients were either obese (n=25; 55.56%) or over weight (n=8; 17.78% ) whereas only 26.7% were normal weight (n=10) or under weight (n=2). We performed PCA scatter plot for visualizing the high dimensional array data. In the scatter plot, each point represents a chip. We applied PCA for identifying outliers and major effects in the data. The results of PCA of the transcriptomic data showed that the samples from the same tissue type clustered tightly together. Clear differences also observed between these cancer and normal tissues revealing distinct expression profiles for the different tissue types (Figure 1). PCA mapping showed that 30% of the overall variance in the microarray dataset is depicted by the first three principal components.

### Identification of differentially expressed genes

**Figure 2:**
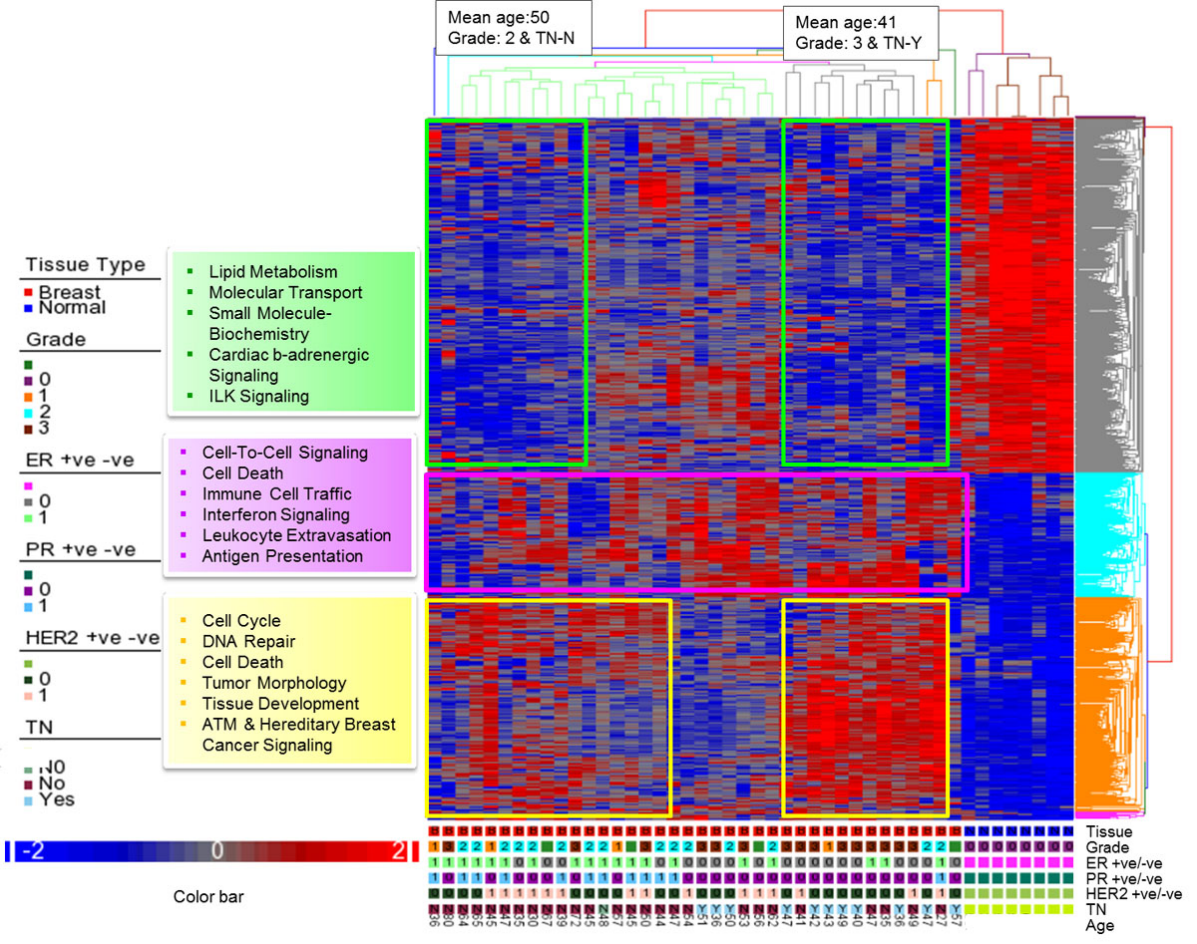
**Functional analysis of altered gene expression data.** Description: Dendrogram shows the change in expression levels of genes in breast cancer compared to normal controls. Samples from each tissue were hybridized to Affymetrix Human ST 1.0 array and signals were scanned after staining and washing the arrays. The data was analyzed as described in the ‘methods’ section, and analysis revealed differential expression of 1159 genes. Agglomerative average-linkage hierarchical clustering for genes (Y axis) and tissue type (X axis) were obtained using Partek GS 6.6 software. The cluster color represents the normalized expression level of a given gene in a particular tissue type or histopathological condition given below and is colored according to the color bar at the bottom. Red denotes upregulation and blue denotes downregulation according the color scale. Each column is single experiment from each subject and each row is a single gene. The data represents 8 controls and 38 breast cancer samples. Seven samples with less information were removed from dendrogram. Yellow, pink and green color boxes indicate cluster of relative differentially regulated genes and their significantly altered functions and pathways. Tumor grade, ER, PR, HER2 and TNBC status were also included in the cluster. ‘1’ denotes +ve status and ‘0’denotes –ve status.

Comparison of the genome-wide expression of breast cancer revealed 1159 differentially expressed genes, 544 genes of which were upregulated and 615 genes were downregulated (2 fold change, false discovery rate p < 0.05). As shown in Figure 2, unsupervised hierarchical clustering revealed that the distinct gene-patterns between the two tissue types. The other sample information including histopathology and age were also overlaid at the bottom of the dendrogram. Cluster analysis also showed that the cancer tissues agglomerated in various subsets according to age, triple negative status and grade. Those of patients with triple-negative breast cancer (TNBC), young age (mean 41) and grade 3 tumor were clearly separated from the old age (mean 50), grade 2 and non-TNBC. We also investigated whether pathway based clustering is evident in the gene signature. For this, genes from each signature were extracted and analyzed for significantly altered biological processes and pathways. Our results suggest that BC patients, despite their histopathologic and age heterogeneity, were clustered into different groups defined by functions and pathways.

Functional analysis of the breast cancer-associated genes found an over expression of genes involved in cell cycle progression, DNA repair, cell death, tumor morphology and tissue developments. Specifically, genes that are known to be associated with BC, including Topoisomerase 2 alpha (*TOP2A*), Carcinoembryonic antigen-related cell adhesion molecule 1 (*CEACAM1*), denticleless/RA-regulated nuclear matrix associated protein (*DTL*), H3 histone family, member A, histone 1, H3a (*HIST1H3A*), the targeting protein for Xklp2 (*TPX2*), Hepatitis C virus NS5A-transactivated protein (*KIAA0101*), centromere protein-F (*CENPF*) and ubiquitin-conjugating enzyme E2T (*UBE2T*) were upregulated compared to the controls. Furthermore, genes associated with immune cell trafficking and interferon signaling including chemokine (*C-X-C* motif) ligand 10 (*CXCL10*), matrix metallopeptidase 11(*MMP11*), interferon, gamma-inducible protein 6 (IFI6), chemokine (C-X-C motif) ligand 9 (*CXCL9*) and matrix metallopeptidase 9 and 13 (*MMP9 and 13*) were also significantly upregulated. Interestingly, the biological process, cellular movement was significantly overrepresented in both down-regulated and up-regulated gene lists pointing that the metastasis was linked to a different equilibrium of switching on and off.

**Figure 3:**
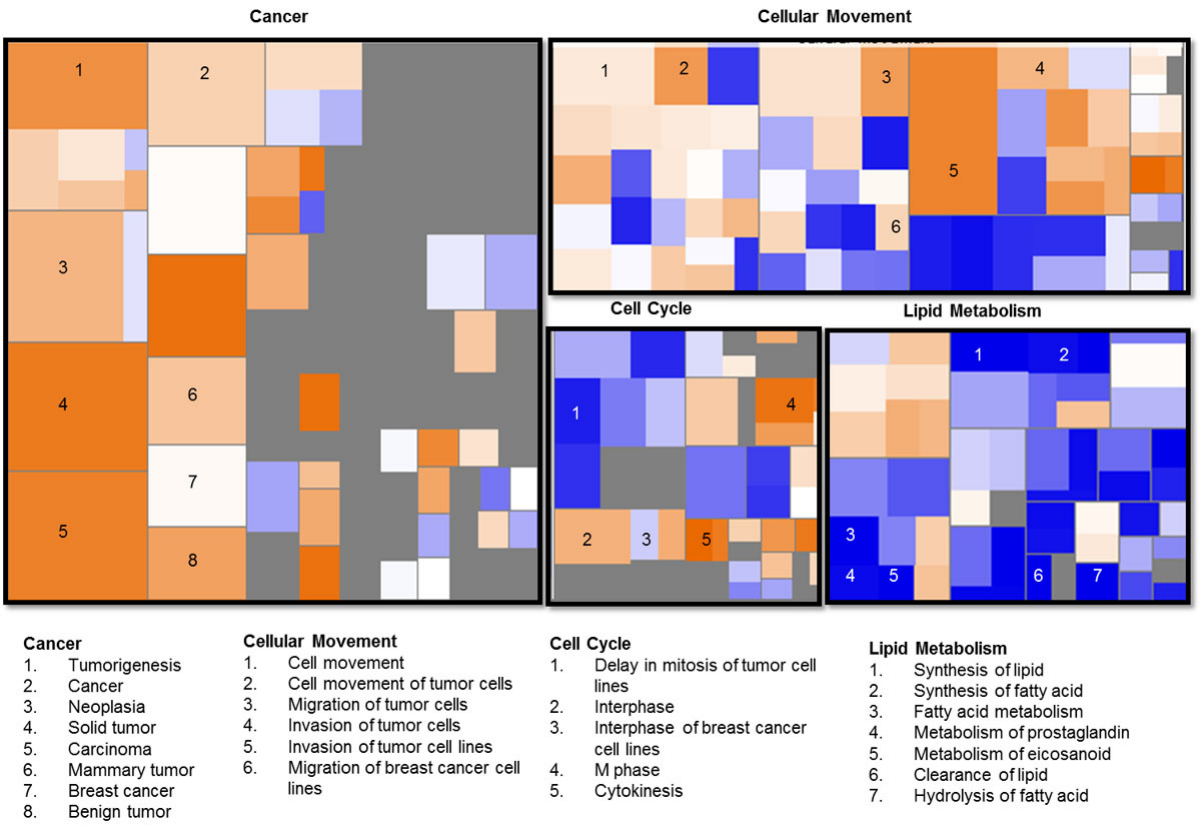
**Molecular and cellular functions deregulated in breast cancer.** Description: Functions are sized by -log (p-value), which shows the associated log of the calculated p-value. Larger squares indicate highly significant overlap between the genes perturbed in the dataset and the biological function or disease. The functions are colored by z-score. The color shows the direction of change for the function, based on the regulation z-score: The positive (orange) score denotes that the biological process or disease is trending towards an increase and the negative (blue) score denotes that the biological process or disease is trending towards a decrease.

Genes linked to lipid metabolism, drug metabolism, and endocrine system development were significantly down regulated in BC. The most downregulated genes including Fatty acid binding protein 4, adipocyte (*FABP4*), adiponectin (*ADIPOQ*), and retinol binding protein 4 (*RBP4*) were known to be involved in lipid metabolism. The downregulated genes also signify biological functions including cardiovascular system development and function, small molecules biochemistry and energy production. Both the functional and canonical pathway analysis indicate that lipid metabolism is one of the most disrupted and downregulated pathways (Table 1; Figure 3).

**Table 1:** Canonical pathways predicted by Ingenuity Pathway Analysis.

Pathways	p-value (Fisher's Exact)	Ratio	Molecules
Glycerolipid Metabolism	6.85E+00	1.36E-01	SDC1 (includes EG:20969),DGAT2,ADH1C (includes EG:11522),LIPE,GK,PNPLA2,ADH1A,GPAM,PPAPDC1A,PPAPDC1B,AADAC,ALDH2,ALDH1A1,PPAP2A,ALDH1A3,AGPAT2,ALDH1A2,LPL,ADH1B,MGLL,PPAP2C

Mitotic Roles of Polo-Like Kinase	5.26E+00	2.15E-01	KIF23,CDC25C,CDC20,PTTG1,PRC1 (includes EG:233406),CDC7 (includes EG:12545),CCNB2,PLK1,CDK1,CCNB1,PLK4,ANAPC5,PPP2R1B,KIF11

Cardiac β-adrenergic Signaling	4.95E+00	1.36E-01	AKAP12,PDE2A,PLN,PPP1R1A,ADRBK2,ATP2A3,PPP1R14A,PDE1A,PDE1C,AKAP2/PALM2-AKAP2,PRKAR2B,GNG11,PDE7B,PDE3B,PDE1B,MRAS,PDE8B,GNG2,PPP1CA,PPP2R1B,APEX1

ATM Signaling	4.70E+00	2.22E-01	RAD51,CDC25C,SMC2,FANCD2,MAPK10,CCNB2,CREB3L4,MAPK13,CREB5,CDK1,CHEK1,CCNB1

ILK Signaling	4.50E+00	1.30E-01	FN1,MYH11,CREB5,RHOH,KRT18,RHOU,FIGF,AKT3 (includes EG:10000),ACTG2,MUC1,CFL1,FERMT2,PIK3C2G,RHOJ,CREB3L4,MYL9,RHOQ,RND3,FLNC,CFL2,ARHGEF6,MAPK10,LEF1,PPP2R1B,MMP9

Protein Kinase A Signaling	3.93E+00	1.03E-01	AKAP12,PHKG2,LIPE,PDE1A,CREB5,HIST2H3C (includes others),PTCH2,TGFBR2,GNG11,PDE7B,PDE3B,PPP1CA,HIST1H1B,APEX1,PDE2A,PLN,GNAI1,PPP1R14A,PYGL,CREB3L4,TTN (includes EG:22138),PDE1C,MYL9,AKAP2/PALM2-AKAP2,PRKAR2B,PYGM,ADD3,FLNC,PDE1B,ANAPC5,PDE8B,LEF1,GNG2,TCF7L2

Leukocyte Extravasation Signaling	3.43E+00	1.15E-01	VAV2,RAC2,CLDN11,JAM2,PIK3C2G,GNAI1,MMP13,MAPK13,CLDN7,DLC1,ITGAL,RHOH,TIMP4,CLDN4,CLDN8,EZR,CXCL12 (includes EG:20315),MAPK10,PECAM1,MMP11,ACTG2,MMP9,CLDN3

Atherosclerosis Signaling	3.11E+00	1.22E-01	CMA1,CD36,MMP13,PLA2G2A,F3,APOC1,IL33,PLA2G4A,ALB,CXCL12 (includes EG:20315),LPL,COL10A1,PDGFD,MMP9,APOD,RBP4

Glycolysis/Gluconeogenesis	2.98E+00	9.85E-02	PGK1,ADH1C (includes EG:11522), ALDH1L1, ALDH2, HK1,ADH1A ,ALDH1A1,ALDH1A3,ALDH1A2,PGM5,ADH1B, ACSL1,ALDOC

Agrin Interactions at Neuromuscular Junction	2.93E+00	1.59E-01	RAC2,PAK3,ARHGEF6,LAMA2,MRAS,MAPK10,ERBB3,ACTG2,ERBB2,ITGAL,EGFR

IL-8 Signaling	2.84E+00	1.04E-01	RAC2,ANGPT1,PIK3C2G,GNAI1,RHOJ,IRAK3,RHOH,CDH1,RHOQ,GNG11,RND3,MAPK10,RHOU,MRAS,AKT3 (includes EG:10000),FIGF,GNG2,MMP9,ITGAX,EGFR

Inhibition of Angiogenesis by TSP1	2.65E+00	1.79E-01	TGFBR2,SDC1 (includes EG:20969),MAPK10,CD36,AKT3 (includes EG:10000),MAPK13,MMP9

Interferon Signaling	2.65E+00	1.94E-01	OAS1,MX1,IRF9,STAT1,TAP1,BAK1,IRF1 (includes EG:16362)

Cell Cycle: G2/M DNA Damage Checkpoint Regulation	2.60E+00	1.63E-01	CDC25C,CKS2,TOP2A,CCNB2,PLK1,CDK1,CHEK1,CCNB1

Cell Cycle Control of Chromosomal Replication	2.52E+00	1.94E-01	MCM3,CDC45,CDC6 (includes EG:23834),CDC7 (includes EG:12545),MCM4,MCM7

Role of BRCA1 in DNA Damage Response	2.47E+00	1.48E-01	RAD51,FANCD2,E2F5,BRCA2,PLK1,BRIP1,STAT1,BLM,CHEK1

Integrin Signaling	2.47E+00	1.00E-01	RAC2,CAPN6,TSPAN7,PIK3C2G,RHOJ,ITGAL,TTN (includes EG:22138), RHOH,MYL9,RHOQ, TLN2,PAK3, RND3,TSPAN1,CAV1,MRAS,RHOU,AKT3 (includes EG:10000), ACTG2 ,ITGA7, ITGAX

RhoGDI Signaling	2.39E+00	9.45E-02	CFL1,GNAI1,RHOJ,DLC1,RHOH,MYL9,CDH2,CDH1,RHOQ,GNG11,PAK3,RND3,EZR,CFL2,ARHGEF6,MRAS,RHOU,ACTG2,GNG2

Production of Nitric Oxide and Reactive Oxygen Species in Macrophages	2.34E+00	9.05E-02	PIK3C2G,PPP1R14A,RHOJ,MAPK13,RHOH,IRF1 (includes EG:16362), APOC1, ALB,RHOQ, RND3,CAT,RHOU,MAPK10,AKT3 (includes EG:10000),STAT1,PPP1CA,PPP2R1B,APOD,RBP4

Hereditary Breast Cancer Signaling	2.29E+00	1.09E-01	CDC25C,HDAC1,PIK3C2G,CDK1,CHEK1,CCNB1,RAD51,FANCD2,MRAS,AKT3 (includes EG:10000),POLR2H,BRCA2,BLM,UBC

Bile Acid Biosynthesis	2.04E+00	7.55E-02	ALDH2,ADH1A,AKR1C1/AKR1C2,ALDH1A1,ALDH1A3,ALDH1A2,ADH1C (includes EG:11522),ADH1B

Estrogen-Dependent Breast Cancer Signaling	1.77E+00	1.14E-01	HSD17B13,IGF1,MRAS,PIK3C2G,AKT3 (includes EG:10000),CREB3L4,CREB5,EGFR

HER-2 Signaling in Breast Cancer	1.21E+00	9.88E-02	CCNE2,MRAS,PIK3C2G,PARD6B,AKT3 (includes EG:10000),ERBB3,ERBB2,EGFR

Breast Cancer Regulation by Stathmin1	7.29E-01	6.67E-02	CCNE2,GNAI1,PIK3C2G,PPP1R14A,CDK1,GNG11,PRKAR2B,ARHGEF6,E2F5,MRAS,TUBA1C,GNG2,PPP1CA,PPP2R1B

The table shows the significantly overrepresented canonical pathways across the whole dataset of differentially expressed genes.

### Pathways and networks underlying breast cancer

To understand the mechanisms by which the genes alter a wide range of physiological processes, we examined molecular networks underlying BC. Transcriptomic signatures showed significant disruption in signaling pathways associating genes of the glycerolipid metabolism, ATM signaling, ILK signaling, DNA damage and cell cycle (Table 1). Analysis by IPA shows a set of key genes that disrupt a pathway such that it then results in tumor initiation or progression. The pathway analysis revealed a strong correlation between the transcriptomic signature and the canonical glycerolipid metabolism which has not been implicated in BC before. Majority of the genes involved in the glycerolipid metabolism were downregulated. These results further support the putative role of these pathways in rendering BC susceptibility.

**Figure 4:**
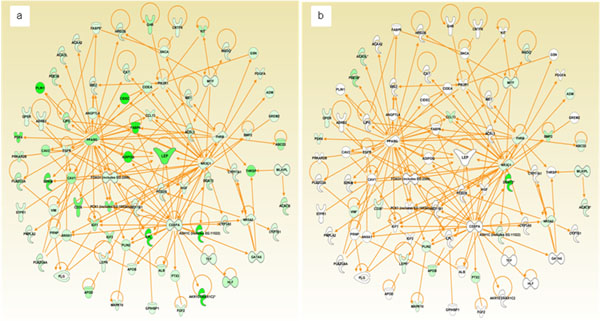
**Comparison of lipid metabolism genes and their expression levels between KSA and USA breast cancer data.** Description: Gene expression data from both the present study as well data from Hawthorn *et al* were imported into IPA. Significantly altered biological functions and pathways were obtained individually for each study. From the differentially expressed genes, those involved in lipid metabolism were linked, and their expression levels were overlaid on to the network. a. KSA data; b. USA data. Molecules colored with green denote downregulation and white denote no change in expression compared to their respective healthy normal.

We further compared the genes involved in lipid metabolism with recent data published from samples from the USA by downloading publically available data of 16 infiltrating ductal carcinoma (IDC) of the breast samples from ArrayExpress accession E-GEO-D-22544(45) (Figure 4). We chose IDC as it accounts for 80% of all breast cancers.

### Therapeutic suggestions using the Connectivity Map

Transcriptomic approaches for studying BC may be useful as it can help to identify pathways that are significantly altered in the specific population or an individual. Moreover, it will allow us to determine specific, effective drugs from the existing database. Having established the feasibility of the computational approaches, we further tried to distinguish compounds that can potentially antagonize the effects of the molecular signature of BC identified in this cohort. Cmap assesses the role of small molecules and genetic effects on transcriptome and establishes links between gene expression signatures and drugs. Based on the connectivity score, we identified 13 selected compounds with average connectivity scores less than -0.7, indicating a high negative correlation (Table 2). Interestingly, five drugs including Fulvestrant, Methotrexate, Letrozole, Doxorubicin and Tamoxifen were FDA approved BC drugs. The compounds that possibly would reverse observed BC signature and hence potential candidates for further *in vitro* validation comprise Hydrocortisone, Gliclazide and Gliclazide. Hydrocortisone and Gliclazide are used for aiding in fat metabolism and as anti-diabetic, respectively.

**Table 2:** Therapeutic suggestions using the Connectivity Map

Pharmaceutical perturbagen	Connectivity score	Description
**Hydrocortisone**	-0.97	Alimentary tract and metabolism (Aid in fat, protein and carbohydrate metabolism)
Fulvestrant *	-0.952	Estrogen receptor antagonist
**Gliclazide**	-0.934	Potent antiatherogenic effect in type 2 diabetes
**Deoxycortone**	-0.917	Corticosteroids for systemic use
**Sirolimus**	-0.909	Antineoplastic and immunomodulating agents
**Glimepiride**	-0.905	Sulfonylurea anti-diabetic drug
**Repaglinide**	-0.895	Treatment of type II diabetes
Methotrexate *	-0.89	Treatment of a number of cancers including breast
Letrozole *	-0.882	Non-steroidal aromatase inhibitor for the treatment of hormonally-responsive breast cancer after surgery
**Glipizide**	-0.878	Anti-diabetic drug from the sulfonylurea class
Doxorubicin *	-0.868	Treatment of a number of cancers including breast
**Glibenclamide**	-0.867	Antidiabetic drug in a class of medications known as sulfonylureas
Tamoxifen *	-0.759	Antagonist of the estrogen receptor in breast tissue

Results of cmap for compounds with expression signatures opposite those of loaded gene expression levels for breast cancer.

* Approved by the FDA for breast cancer

## DISCUSSION

Breast cancer therapeutic strategies today are generally based on histopathological characterization, tumor size, grading, and axillary lymph node and receptor status [1]. Even so, patients when diagnosed with similar conditions and when treated with similar drug can go through extensive differences in the development and relapse of BC. Studies show that women, who had a full-term pregnancy at an age below 30; and who went through three full-term pregnancies, and three or more years of breast feeding, were significantly protected against BC [20]. However, BC incidence is high among women from the KSA. BC risk factors including nulliparity or low parity, first full term pregnancy at very late age, no breast feeding etc., are not common in the Saudi society [19]. These factors combined with the early onset of BC among women, in this ethnicity, prompted us to study the molecular mechanism underlying the malignancies.

In the present study, we identified transcriptomic signature in BC from the KSA that is associated with clinical and histological parameters. The ethnicity of this cohort renders the power to prospectively examine, the roles of lifestyle and genetic susceptibility in the onset of BC. The genome-wide expression analysis contains several overrepresented functional gene classes and has substantial overlaps with transcriptomic signatures of metastatic human BC. The commonalities found between different populations in terms of increased cell cycle regulation and DNA integrity checkpoints were observed. A similar transcriptomic signature for many of these genes has been reported for human BC before [22-26].

Genes associated with lipid metabolism and small molecule biochemistry was significantly downregulated in the BC tissues. The role of these genes on the tumorigenesis of BC has been reported previously [27-29]. Disruption of lipid metabolism has been implicated in tumorigenesis in several studies, especially in cancer of breast [30,31]. The differential regulation of lipid metabolism between normal and cancer subjects might reveal transformation in the metabolism of the cancer patient in this ethnicity [31]. Decreased *ADIPOQ* levels interrupt cellular signaling networks that are linked to cell survival, angiogenesis, proliferation, and cell-cycle regulation [32]. Adipose tissues secrete *ADIPOQ and LEP*-*ADIPOQ* axis has been well implicated in breast cancer tumorigenesis [32]. Levels of ADIPOQ have been inversely correlated with obesity. Interestingly, studies show that women with increased *ADIPOQ* concentrations possess 65% reduced risk for breast cancer [27]. In another study, it has been shown that patients with low *ADIPOQ* levels had a significantly increased likelihood of cancer recurrence.

Unsupervised clustering analysis also revealed the gene expression signatures to be associated with histologic grade, age and triple negative status. This signature could also be used for improving stratification of patients with BC in this population. Further pathway analysis of differentially regulated genes provides novel hypotheses underlying metastatic progression of BC. In addition, cmap analysis can formulate novel therapeutics for BC, starting from a cellular approach to investigating the effects of the compound determined by cmap. It may be possible to develop stratified therapy by combining both standard chemotherapy and pathway-specific therapies.

*ADIPOQ*, an adipocyte secreted protein, was found to one of the most downregulated gene in our analysis and it’s lower expression levels have been shown to be associated with obesity, insulin resistance, and type 2 diabetes mellitus [33]. It has also been shown to be reduced in pre-menopausal women with endometrial cancer, which is tightly linked with obesity and insulin resistance. A case control study found significantly inverse relationship of serum *ADIPOQ* with BC [34].

Comparison of BC data from USA with KSA further revealed distinct expression levels for *LEP* and other genes involved in lipid metabolism [35]. In our analysis, glycerolipid metabolism was found to be a highly significantly altered canonical pathway. However, comparative analysis of these findings is different from the recent transcriptomic profiles of BC from the USA data, as glycerolipid metabolism was ranked 41^st^ in the USA data [35]. In the KSA samples, glycerolipid metabolism was one the most overrepresented pathways, whereas, PPARα / RXRα activation pathway is the most disrupted one in the USA data.

To comprehend population differences in the severity of BC, studies linking both genetic and environmental factors and examining cases from different populations are important. Researchers have been investigating issues surrounding lifestyle, circadian rhythm, obesity, and adverse exposures in an attempt to identify susceptibility factors in initiating BC. Since diet and obesity contribute to changes in circulating hormone levels, they both play a key role in the development of BC. Understanding the underlying genetics and complex interactions of lifestyle, diet, and other known risk factors will remain a key area of research.

The prevalence of obesity among Saudi patients adults was 55.56% in comparison to 33.8% in USA adults, however combined obese and overweight percentage were almost same with 68.0% in western and 73 % in Saudi BC patients [36]. Obesity is primarily characterized by excess fat storage, adipocyte mass, and increase in certain types of lipids. Normally healthy women have 14–28% of fat mass in the body, but it may increase up to 60–70% in morbidly obese individuals [37]. Earlier white adipose tissue function was assigned for purely an energy storage tissue, however, recent finding has uncovered the endocrine and metabolic properties that has led to several mechanisms implicated in how obesity drives cancer prevalence and cancer deaths. Many possible mechanisms have been proposed to explain the increased risk of breast cancer associated with obesity such as increased lipids and lipid signaling, inflammatory responses, insulin resistance, adipokines, altered immune responses, and oxidative stress. Study had shown that cancer cells can access lipids from neighboring adipocyte stores and can directly use these transferred lipids as an energy source, which in turn promotes tumor growth [38]. Thus, it is critical to understand the physiological impact of obesity on breast cancer development and progression. In recent years, obesity has been identified as a significant modifiable increased risk factor for breast cancers among postmenopausal women but is unrelated or inversely related to risk among premenopausal women not in pre-menopausal women [39-43]. A study examined obesity and mortality from cancer in 495,477 US women, and found that obese women had double the death rate (relative risk, 2.12) than women with normal BMI (25 or less) [44].

Fat tissue of obese women are most important source of estrogen after ovary, secreting higher level of estrogen potentially leading to more rapid growth of estrogen-responsive breast tumors [45]. Fat cells may also promote inflammation linked cancer through tumor growth regulators and tumor necrotic factors. Obesity often cause insulin resistance, which may promote the development of breast cancer [46,47]. Adipose tissue secrete TNF-α leading to inflamation promoting cancer. Fat cells produce hormones, called adipokines, that may stimulate or inhibit cell growth [48]. For example, adiponectin, a down regulated gene in present study, has been reported to be inhibitor for proliferetion and negative regulator of angiogenesis [37,49,50]. Further a significant inverse correlation between serum adiponectin levels and poor-prognosis breast cancer were confirmed. Thus, low adiponectin in obese individuals could increase risk of developing tumors with aggressive angiogenesis [34,51].

## Conclusions

This study described, to our knowledge, the first genome-wide profiling of BC from Saudi ethnic females. Our analysis reveals appropriate biological relevance and a number of molecular pathways that may serve as targets for novel therapeutics. Our finding of 73.3% obese vs 26.7% normal weight clearly suggest that obesity increases the risk of breast cancer occurrence. Further studies are needed to determine the relationship between lipid metabolism dysregulation and the mechanisms underlying BC. This study may open new avenues of experimental strategies for further examination in larger cohorts of all subtypes, correlating ethnicity to molecular signature and life-style.

## METHODS

### Patients and samples

The study was performed on female BC patients from the KSA, diagnosed with invasive ductal carcinoma. The samples were collected from one of the three participating Medical Centers in Jeddah, the KSA including King Abdulaziz University Hospital, King Faisal Specialist Hospital and Research Center, and Bakshs Hospital, during years 2008-2011. For gene expression analysis, fresh tumor tissue specimens were cut from fresh surgical resections adjacent to the sites on which final histological diagnosis was performed. Fresh normal breast specimens were derived from surgically resected normal breast tissues. Patients excluded from this study had at least one of the following exclusion criteria: histopathological diagnosis was not invasive ductal carcinoma; patient history, and medical files, or specimens were not found. This left samples from 45 tumors available for transcriptomic analysis. All collected tissue specimens were immediately placed in RNALater (Invitrogen - Life Technologies, Grand Island, NY) or RPMI 1640 medium (GIBCO, USA). Clinicopathological features such as age, tumor grade, tumor size, hormone receptor status, lymph node involvement and pathology reports were retrieved from the patients’ records after obtaining all the relevant ethical approvals. The complete histoclinical characteristics of the 45 BC patients are summarized in Table 3. The average age in the present cohort was 48 years, with a median of 47 years (range between 27-80 years). Body mass index (BMI), a measure of weight relative to height was used to estimate Normal weight (≥18 -24.9 kg/m^2^ overweight (≥25-29.9 kg/m^2^) and obesity (≥30 kg/m^2^).

**Table 3:** Clinicopathological characteristics of the 45 breast cancer patients.

Characteristics	N	%
Sex		

Female	45	100

Age		

≤ 50	32	71

≥50	13	29

Age, mean (range) years	48 (27-80)	

BMI (Kg/m^2^)		

Obese (≥30 Kg/m^2^)	25	56

Over weight (≥25-29.9 Kg/m^2^)	8	18

Normal weight (≥18- 24.9 Kg/m^2^)	10	22

Under weight (≤18 Kg/m^2^)	2	4

Site		

Right	25	56

Left	18	40

NA	2	4

Histological type		

IDC	34	76

ILC	5	11

Mixed	2	4

Others	2	4

NA	2	4

Tumor size: mean (sd) (cm)	3.1 (±1.4)	

Grade		

1	4	9

2	17	38

3	17	38

NA	7	16

pN		

Negative	19	42

Positive	23	51

NA	3	7

LI		

Negative	17	38

Positive	19	42

NA	9	20

ER (IHC)		

Negative	19	42

Positive	19	42

NA	7	16

PR (IHC)		

Negative	27	60

Positive	11	24

NA	7	16

HER2 (IHC)		

Negative	24	53

Positive	14	31

NA	7	16

TNRS		

Yes	11	24

No	34	76

N: number of cases; BMI: Body Mass Index; NA: information not available; IDC: invasive ductal carcinoma; ILC: invasive lobular carcinoma; pN: pathological lymph node involvement; LI: lymphatic invasion; ER: estrogen receptor; IHC: immunohistochemistry; PR: progesterone receptor; TNRS: Triple-negative receptor status

### Ethical approval

All patients included in the study provided written informed consent. The study was reviewed and approved by the Center of Excellence in Genomic Medicine Research (CEGMR) ethical committee (approval number 08-CEGMR-02-ETH).

### RNA extraction and array processing

Total RNA was extracted from fresh breast tissue specimens with the Qiagen RNeasy Mini Kit (Qiagen, Hilden, Germany) including an on-column DNAse treatment according to manufacturer’s recommendations. Quality of the purified RNA was verified on an Agilent 2100 Bioanalyzer (Agilent Technologies, Palo Alto, CA). Mean value of RNA integrity number (RIN) for all 50 processed samples was 8.0. RNA concentrations were determined using a NanoDrop ND-1000 spectrophotometer (NanoDrop Technologies, Wilmington, DE). 300 ng of each RNA sample was processed according to the manufacturer`s recommendation (Affymetrix, Santa Clara, CA, USA). After fragmentation and labeling, the samples were hybridized at 45°C for 17 hours to Human Gene 1.0 ST GeneChip arrays (Affymetrix, Santa Clara, CA, USA). These arrays are conceptually based on the Human Genome sequence assembly UCSC hg18, NCBI Build 36 and interrogate with a set of 764,885 probes 28,869 annotated genes.

### Gene Expression Analysis

Affymetrix .CEL files and were imported to Partek Genomics Suite version 6.5 (Partek Inc., MO, USA). The data was normalized using RMA normalization. Principal component analysis (PCA) was performed on all probes to visualize high dimensional data. PCA was used to assess quality control as well as overall variance in gene expression between the disease states. Analysis of Variance (ANOVA) was applied on the complete data set and differentially expressed gene list was then generated using an FDR (Benjamini Hochberg) of 0.05 with 2 fold change cut off. Unsupervised two dimensional average linkage hierarchical clustering was performed using Spearman’s correlation as a similarity matrix. The microarray data generated in this study are in compliance with MIAME (http://www.mged.org/Workgroups/MIAME/miame.html) guidelines.

### Functional and Pathway analysis

To define biological networks, interaction and functional analysis among the differentially regulated genes in BC, pathway analyses were performed using Ingenuity Pathways Analysis software (IPA) (Ingenuity Systems, Redwood City, CA). Statistically differentially expressed dataset containing 1159 genes and their corresponding probesets ID, Gene symbol, Entrez gene ID as clone identifier, p-value and fold change values were uploaded into the IPA. The functional/pathway analysis of IPA identifies the biological functions and/or diseases and pathways that are most significantly altered for the differentially expressed gene set. The significance of the connection between the expression data and the canonical pathway were calculated by ratio and/or Fisher’s exact.

### Connectivity Map analysis

The differentially expressed genes identified were analyzed using the cmap in an effort to link genes associated with a phenotype with potential drug molecules. Cmap analyzes the correlation between our gene expression signature and predefined signatures of therapeutic compounds. A ‘‘connectivity score” (+1 to -1), signifying relative similarity to our differentially expressed genes, was generated using a metric based on the Kolmogorov-Smirnov statistic, as described [21]. A compound with negative connectivity scores represents compounds that potentially would reverse the molecular phenotype.

### Availability of supporting data

The clinicopathological information and datasets (.CEL file) supporting the results of this article were submitted to NCBI’s Gene Expression Omnibus (GEO) under accession number GSE36295. (http://www.ncbi.nlm.nih.gov/geo/).

## Conflicts of interest

The authors declare that they have no potential conflicts of interest.

## Authors' contributions

AM, SK, AD, FA and MHA participated in the study design, interpretation of the results and finalization of the manuscript. HJS conducted microarray and AB conducted pathological studies. SK and JM analyzed data and drafted the manuscript. MG, AC, and AA participated in data collection, editorial support and critical review of manuscript. All authors read and approved the final manuscript.

## Declarations

Publication charges for this article have been funded by Center of Excellence in Genomic Medicine Research, Faculty of Applied Medical Sciences, King Abdulaziz University, Jeddah, Saudi Arabia

This article has been published as part of *BMC Genomics* Volume 16 Supplement 1, 2015: Selected articles from the 2nd International Genomic Medical Conference (IGMC 2013): Genomics. The full contents of the supplement are available online at http://www.biomedcentral.com/bmcgenomics/supplements/16/S1
